# Uneven Index: A Digital Biomarker to Prompt *Demodex* Blepharitis Based on Deep Learning

**DOI:** 10.3389/fphys.2022.934821

**Published:** 2022-07-11

**Authors:** Xinyi Liu, Yana Fu, Dandan Wang, Shoujun Huang, Chunlei He, Xinxin Yu, Zuhui Zhang, Dexing Kong, Qi Dai

**Affiliations:** ^1^ School of Ophthalmology and Optometry, Eye Hospital Wenzhou Medical University, Wenzhou, China; ^2^ Department of Ophthalmology, People’s Hospital of Yichun, Yichun, China; ^3^ Department of Ophthalmology, Second Affiliated Hospital, School of Medicine, Zhejiang University, Hangzhou, China; ^4^ Department of Ophthalmology, The First Affiliated Hospital of Soochow University, Suzhou, China; ^5^ College of Mathematical Medicine, Zhejiang Normal University, Jinhua, China

**Keywords:** artificial intelligence, *Demodex* blepharitis, digital biomarker, total variation, uneven atrophy

## Abstract

**Purpose:** To evaluate ocular surface manifestations and morphological changes in meibomian glands (MGs) based on artificial intelligence (AI) analysis in patients with *Demodex* blepharitis.

**Methods:** In this retrospective study, 115 subjects were enrolled, including 64 subjects with *Demodex* blepharitis and 51 subjects without *Demodex* blepharitis as control group. Morphological indexes were evaluated for height, width, tortuosity, MG density, total variation, and the three types of corrected total variation as Uneven indexes.

**Results:** There were no statistically significant differences in all MGs’ average tortuosity and width between the two groups. The average height of all MGs and MG density were significantly lower in the *Demodex* blepharitis group than control group. The total variation and two types of Uneven indexes were significantly higher in the *Demodex* blepharitis group than in the control group. Especially the Uneven Index of total variation/MG density had an AUC of 0.822. And the sensitivity and specificity were 59.4% and 92.2%, respectively, at a cut-off value of 3971.667. In addition, *Demodex* blepharitis was associated with significantly lower meibum quality and expressibility, severe atrophy of MGs, a higher ocular surface disease index (OSDI), and more instability of the tear film.

**Conclusion:** *Demodex* mites are strongly associated with morphological changes in the MGs and may cause uneven gland atrophy. Therefore, the novel characteristic parameter, the Uneven index, may serve as a digital biomarker to evaluate uneven atrophy of MGs and prompt *Demodex* blepharitis.

## 1 Introdution

*Demodex* mites are the most common permanent ectoparasites in humans (Basta-Juzbasić et al., 2002). *Demodex folliculorum* was discovered as early as 1841 in humans, and in 1875 the follicle mite was found in the excretory duct of a meibomian gland (MG) for the first time (Rabensteiner et al., 2019). It has been identified as a vector for different bacteria, fungi, and viruses in disease development and is associated with chalazia, blepharitis, conjunctivitis, corneal manifestations, meibomian gland dysfunction (MGD) and dry eye (Demmler et al., 1997; Morfin Maciel et al., 2003; Kheirkhah et al., 2007; Liang et al., 2010; Hao et al., 2022).

Several studies have demonstrated a strong correlation between ocular demodicosis and MGD (Randon et al., 2015; Alver et al., 2017; Zhang et al., 2018; Yan et al., 2020). These results indicated that the severity of ocular *Demodex* infestation could aggravate the symptoms of eye discomfort, such as eye itching, burning, and dryness. Ocular demodicosis greatly impacts the change of MG morphology (Gao et al., 2005; Kheirkhah et al., 2007; Lee et al., 2010). Some studies found that demodicosis was associated with severe MG atrophy that presented with more than one-third MG loss (Liang et al., 2013; Liang et al., 2018). In addition, Cheng et al. (2019) also indicated that *Demodex* infestation could cause microstructural changes in MGs, such as MG acinar longest diameter enlargement, MG fibrosis and reduced acinar density, MG acinar irregularity, and inhomogeneous appearance of aggravated walls of acinar units. However, studies on the morphological changes of MGs caused by *Demodex* infestation are scarce, and the relationship between morphological changes of MGs and *Demodex* infestation has not been quantitatively examined.

As an effective diagnostic tool for quantifying MG morphological features obtained using meibography, the artificial intelligence (AI) system based on a convolutional neural network (CNN) allows accurate observation of each MG. Some AI system allows automatic analysis of the morphological indexes of MGs in detail, such as their height, width, tortuosity, and density (Dai et al., 2021). Therefore, the AI system based on CNN was employed to observe the morphological changes of MGs in an attempt to quantitatively examine the role of *Demodex* infection in the ocular changes of MGD.

In addition, in the clinic, we found that compared with common MGD patients, MG atrophy is more uneven in patients with hordeolum stye and chalazion, especially in patients with *Demodex* blepharitis. However, there is presently no specific quantitative index to verify this phenomenon. Therefore, in our study, the “Uneven index” was introduced as a novel evaluation index to verify the uneven morphological characteristics of the MGs better.

In this study, we compared the differences in clinical features and morphological changes of MGs between outpatients with *Demodex* blepharitis and control group based on a deep learning model to evaluate the Uneven index as a novel digital biomarker for *Demodex* blepharitis.

## 2 Materials and Methods

### 2.1 Participants

This is a retrospective study. All data were collected from June 2019 to September 2020 at the Affiliated Eye Hospital of Wenzhou Medical University, China. A total of 115 subjects (8–78 years old) were included in the study; Only one eye of each subject was randomly selected and included. Among them, 64 subjects were diagnosed with *Demodex* blepharitis, and the other 51 subjects without *Demodex* infestation and blepharitis were randomly selected and included as the control group.

The exclusion criteria were as follows: 1) a history of ocular trauma or surgery; 2) systemic drugs or eye drops affecting MG function or tear film used in the last 2 weeks; 3) contact lenses worn in the last 2 weeks; and 4) other ocular or systemic diseases affecting MG function or tear film.

Patients with MG density below 0.05 were removed to reduce the errors caused by the extreme atrophy of MGs. When the MG density was very low, and gland atrophy was severe, each index of MG was close to 0, and the evaluation efficiency of MG morphology was lost.

This study was performed in accordance with the Declaration of Helsinki and approved by the Research Ethics Committee of the Eye Hospital, Wenzhou Medical University (approval number: 2020-209-K-191). Written informed consent was obtained from all study patients. This study was registered at http://clinicaltrials. gov (NCT04451122).

### 2.2 Diagnosis of *Demodex* Blepharitis

*Demodex* blepharitis was diagnosed based on the diagnostic criteria (at least one symptom, such as redness, eye itching, foreign body sensation, abnormal eyelashes with cylindrical dandruff, and positive results on light microscopic examination of the eyelashes) (Amescua et al., 2019). *Demodex* mites were detected and counted by a professional technician. Three eyelashes were pulled from each eyelid using forceps under a slit-lamp microscope. The eyelashes from each eyelid were placed separately parallel to a glass slide, covered with a cover slide, and the *Demodex* mites were counted under a light microscope. Each glass slide was dropped with 0.25% fluorescein, with added 20 μl of cedar oil and 100% ethanol when there were too many scales in the root of the extracted eyelashes to improve detection and counting of mites. Patients were diagnosed with *Demodex* positive if 1) *Demodex* mites in each stage (mites whether dead or alive, larvae, and eggs) were observed; 2) more than three mites were found on any eyelid; and 3) less than the above standards may have been diagnosed as a suspicious infestation, which needed to be combined with clinical manifestations (Asian Dry Eye Association China Branch, 2018).

### 2.3 Image Acquisition and Analysis Using Artificial Intelligence System Based on Convolutional Neural Network

Meibography images were captured with a K5M (Markoulli et al., 2017). Then, the MGs from these images were extracted using a novel MG extraction method based on CNN with a residual neural network, which was introduced in our previous study (Zhang et al., 2022).

We replaced the 50-layer ResNet (ResNet50) with the max-pooling layers of the U-net model; however, the up-sampling layer remained the same, and this model was called ResNet50_U-net. For model training, the meibography images were firstly optimized, converted to grayscale, and then standardized and normalized (Dai et al., 2021). Forty annotation meibography images were included as the basis for the training set. Meanwhile, the data enhancement model, imgaug (https://github.com/aleju/imgaug#citation, 6 February 2020), was used to generate new training images from randomly selected original annotation meibography images. In this ResNet50_U-net model, the batch size of each iteration was four, so imgaug generated four random new images each time to form a mini batch. A complete training consisted of 120 epochs, while each epoch consisted of 512 iterations. Thus, it contained a total of 61,440 iterations, using 245,670 training images generated by imgaug. Another 20 annotated original meibography images apart from the training set were used as the validation set.

Intersection of unions (IoU) was used to evaluate the accuracy of the MG segmentation model. It can be simply understood as the ratio of the intersection of the ground truth (manual annotation) and AI result (AI segmentation) to their union. Finally, the ResNet50_U-net model achieved 92.00% in IoU, and 100% in repeatability.

**FIGURE 1:**
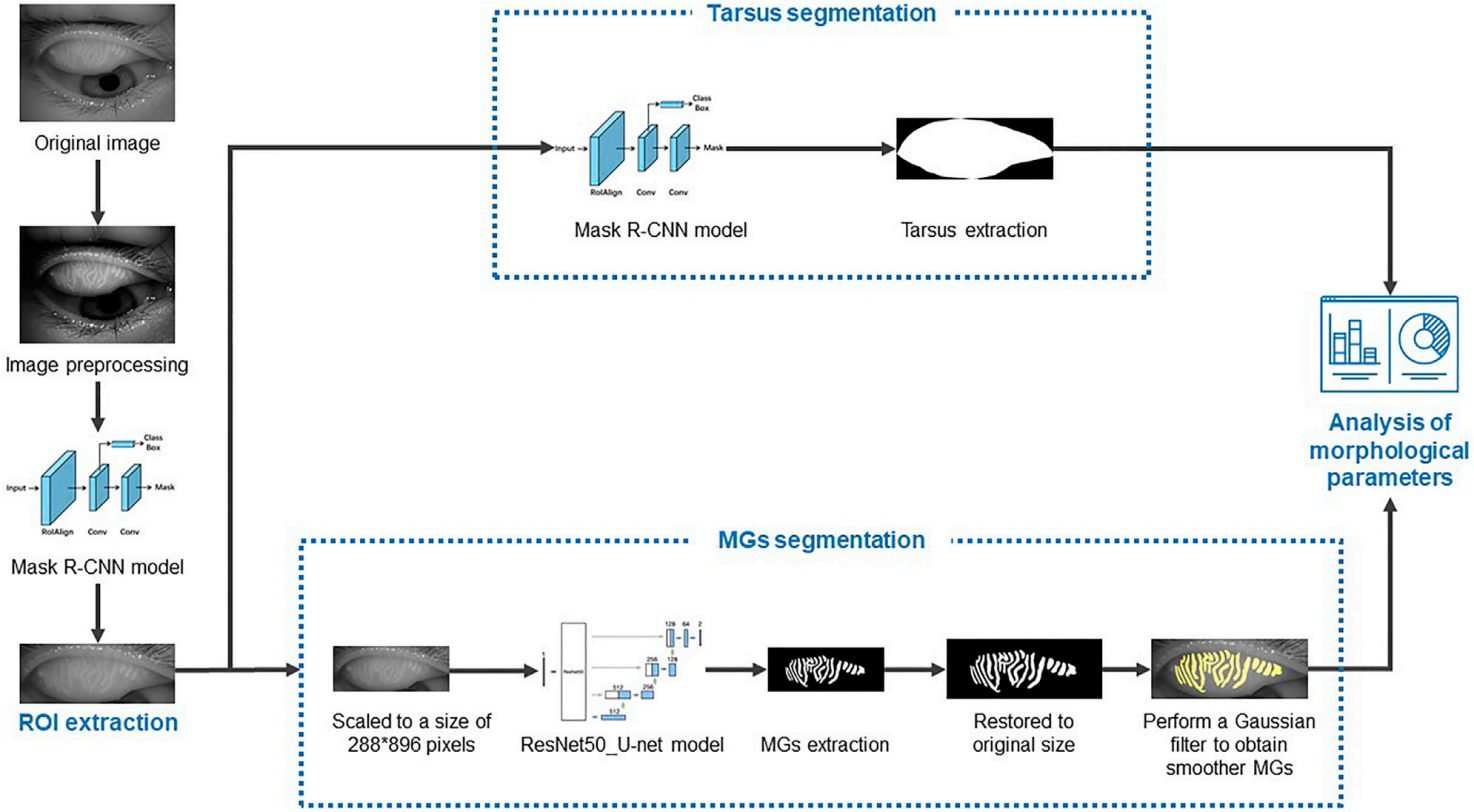
The flowchart of image processing and segmentation.

The flowchart of image processing and segmentation is shown in Figure 1.

### 2.4 Morphology Indexes

After extracting the MGs, the following indexes were automatically calculated with the aid of a computer (Zhang et al., 2022).

#### 2.4.1 Height and Width of Each Meibomian Gland

The height was the length of the minimum external rectangle of the MG, and the width was the area divided by the height.

#### 2.4.2 Meibomian Gland Tortuosity

The tortuosity was calculated by the formula below.

$$MG\;tortuosity=\frac{MG\;perimeter}{2\times height\;}-1$$

#### 2.4.3 Meibomian Gland Density

The sum of the area of MGs divided by the total area of the tarsus in pixels. $\overset{n}{\underset{i=1}{\sum }}S_{MG_{i}}$ = the sum pixels of all MGs, *St* = the total pixels of the tarsus.

$$MG\;density\;=\;\frac{\sum_{i=1}^{n}S_{MG_{i}}}{St}$$

#### 2.4.4 The Total Variation

The total variation of a function with one variable is used to measure the magnitude of its oscillations, indicating its uneven properties. Generally, the larger the total variation, the more uneven the MG dystrophy. For a function f(x) defined on an interval [a,b] , the total variation is given by:

$$\overset{b}{\underset{a}{\text{V}}}f\left(x\right)=\sup\left\{\sum_{j=1}^{n}|f\left(x_{j}\right)-f\left(x_{j-1}\right)|\right\},$$

where the supremum is taken over all finite partitions

$$\left\{a=x_{0}<x_{1}<\cdots <x_{n}=b\right\}$$

If $\overset{b}{\underset{a}{\text{V}}}f\left(x\right)$ is finite, then f(x) has bounded variations. The bounded variation is defined differently for a function with several variables, which is often assumed to be Lebesgue measurable. However, it is essentially equivalent to the above definition if we assume that the space dimension of the function is equal to one (Evans and Gariepy et al., 1992). Nowadays, the bounded variation space, consisting of functions with bounded variation, has wide applications in image processing (Aubert and Kornprobst, 2006).

In this study, the total variation refers to the addition of the absolute value of the difference in the height of each adjacent two glands in the meibography images.

#### 2.4.5 Uneven Index

Under the condition of different gland numbers or MG densities, the unevenness represented by the same total variation was inconsistent. When there are fewer glands or more severe atrophy, a smaller total variation may represent greater unevenness. We used multivariate regression analysis to analyze the indexes that may affect total variation and used related indexes to correct for total variation. We then obtained the mean total variation, namely the Uneven index, as shown below:

##### 2.4.5.1 Uneven Index 1

Total variation/N = total variation/gland number.

##### 2.4.5.2 Uneven Index 2

Total variation/D = total variation/MG density.

### 2.5 Clinical Parameters

All participants underwent the ocular surface disease index (OSDI) questionnaire (Markoulli et al., 2017), slit-lamp examination and ocular anterior segment photography. The ophthalmic examinations included the following: 1) tear meniscus height (TMH): the height was measured 5 s after blinking, and the central TMH of the lower eyelid was measured. 2) Tear film break-up time (TBUT): The time before the first defect appeared on the stained tear film was measured as the TBUT after fluorescein instillation (repeated three times, and the mean value was recorded). 3) Corneal fluorescein staining (CFS): CFS was performed after the installation of fluorescein, and the degree was graded using the Baylor grading scheme from 0 to 4. 4) Lid margin score including anterior or posterior displacement of the mucocutaneous junction, vascular engorgement, plugged meibomian gland oriflces, and irregularity of the lid margin. It was scored from 0 to 4 according to the four aforementioned parameters. 5) Meibum expressibility score: The function of the 15 glands on each lower eyelid was assessed. The secretion for each gland was graded as follows: 0 = no secretion, 1 = inspissated (toothpaste consistency), 2 = cloudy, and 3 = clear-to-normal secretion. The scores ranged from 0 to 45 (Cochener et al., 2017).

### 2.6 Statistical Analysis

The normality of data distributions was analyzed using the Kolmogorov–Smirnov test, and abnormal data distributions were analyzed using non-parametric statistical analyses. Values are expressed as the mean ± standard deviation (SD) or median [interquartile range (IQR)], and the range of data. The independent samples *t*-test or Mann–Whitney U-test was used to compare differences between the two groups. A generalized estimating equation was used to adjust for age differences. The χ2 test was used to compare sex ratios between the two groups. Receiver operating characteristic (ROC) curve analysis was used to determine the predictive value of total variation in the diagnosis of MGD with Demodex infestation. Multivariate regression analyses were used to evaluate the relationship between the total variation and MG parameters. A two-sided *p* < 0.05 was considered statistically significant. All statistical analyses were performed using SPSS Statistics (version 23.0; IBM, Armonk, NY, United States).

## 3 Results

### 3.1 Basic Characteristics and Ocular Surface Manifestations

In this retrospective study, the records of 115 subjects were analyzed at the dry eye department, Eye Hospital Wenzhou Medical University, Zhejiang, China. Of these, 64 subjects diagnosed with *Demodex* blepharitis were in the *Demodex* group, and 51 subjects without *Demodex* blepharitis were in the control group. The basic characteristics and clinical parameters are shown in Table 1.

**TABLE 1:** Basic characteristics and the clinical parameters of the subjects in *Demodex* group and control group.

Parameters	*Demodex* group (*n* = 64)	Control group (*n* = 51)	*p v*alue	*p v*alue*
Age (years)	39.0 (28.0, 57.0)	28.98 ± 11.46	<0.05	—
(Range)	(19–78)	(8–57)		
OSDI (0–100)	22.9 (13.0, 41.7)	7.1 (2.5, 23.2)	<0.001	0.002
(Range)	(0–100.0)	(0–79.6)		
Tear meniscus height (mm)	0.2 (0.2, 0.2)	0.19 ± 0.06	0.104	0.086
(Range)	(0.13–0.42)	(0.06–0.33)		
Tear film break-up time (seconds)	2.3 ± 1.3	5.0 (2.2–5.0)	<0.001	<0.001
(Range)	(0–5)	(1–20)		
Corneal fluorescein staining (0–20)	0.0 (0.0–2.0)	0.0 (0.0–0.0)	0.138	0.587
(Range)	(0–8)	(0–4)		
Lid margin score (0–4)	2.0 (2.0, 3.0)	1.0 (0.0, 2.0)	<0.001	<0.001
(Range)	(0–4)	(0–2)		
Meibum expressibility score (0–45)	8.0 (4.0, 14.0)	31.5 (9.5, 42.0)	<0.001	<0.001
(Range)	(0–29)	(0–45)		
Meiboscore (0–6)	3.0 (2.0–4.0)	2.0 (1.0–3.0)	<0.001	0.001
(Range)	(0–6)	(0–5)		

*p values adjusted for age by generalized estimating equation. OSDI, ocular surface disease index.

Participants in the *Demodex* group were significantly older [39.0 (28.0–57.0) years]) than those in the control group (28.98 ± 11.46 years), so the analysis of covariance was used to adjust for age for the comparisons between the two groups. Significant differences were noted in the OSDI, TBUT, lid margin score, meiboscore, and meibum expressibility scores between the two groups (all *p* < 0.05; Table 1). No difference was noted in the TMH (*p* = 0.086), and CFS (*p* = 0.587).

### 3.2 *Demodex* Morphological Indexes Between Two Groups

Table 2 shows the analysis results of the morphological changes in MGs in the *Demodex* and control groups. Compared to the control group, patients in the *Demodex* group had significantly lower densities and shorter heights of all MGs in the upper eyelid (all *p* < 0.05). However, there were no statistically significant differences in the average tortuosity (*p* = 0.426), width (*p* = 0.557), and gland number (*p* = 0.885) between the two groups.

**TABLE 2:** Upper lid MG morphological changes in *Demodex*-positive and *Demodex*-negative patients.

Parameters	*Demodex* group (*n* = 64)	Control group (*n* = 51)	*p* value	*p* value*
Total variation	927.3 ± 343.1	650.0 (569.5, 865.5)	0.003	0.004
(Range)	(315.0–1698.0)	(152.0–1725.0)		
Average tortuosity of all MGs	0.4 (0.3, 0.5)	0.3 (0.3, 0.5)	0.173	0.426
(Range)	(0.14–0.91)	(0.12–0.83)		
Average width of all MGs	22.58 ± 4.58	22.7 (20.5, 25.5)	0.206	0.557
(Range)	(13.02–35.36)	(16.00–34.88)		
Average gland number	16.05 ± 3.86	16.31 ± 3.88	0.714	0.885
(Range)	(6–48)	(9–27)		
Average height of all MGs	157.39 ± 42.43	227.83 ± 47.24	<0.001	<0.001
(Range)	(78.53–251.76)	(86.89–309.64)		
MG density	0.22 ± 0.07	0.30 ± 0.09	<0.001	0.008
(Range)	(0.06–0.42)	(0.07–0.47)		

p-value of Mann–Whitney U test or independent-t test for continuous parameters and of χ2 test for categorical parameters; MG, meibomian gland; *p values adjusted for age by analysis of covariance

The total variation was significantly higher in the *Demodex*-positive group than in the *Demodex*-negative group (*p* = 0.004).

**FIGURE 2:**
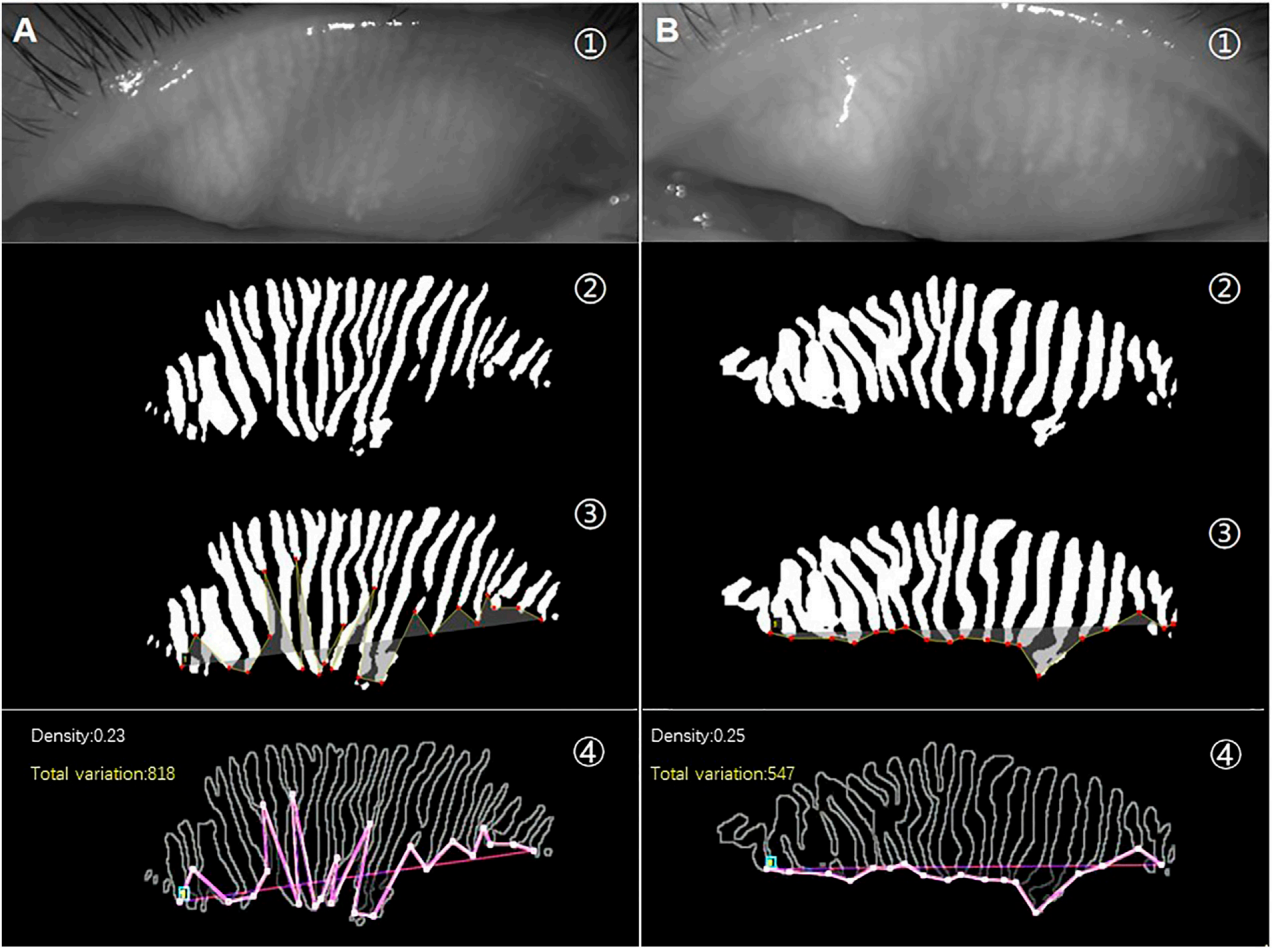
**(A)** is a meibography image from a patient with *Demodex* blepharitis, and **(B)** is a meibography image from the control group. The raw images (①) and AI prediction results of the same image (②). Lines formed by connecting the ends of all MGs in the same image are shown in yellow (③). An obvious difference in the pink lines represents total variation between the two meibography images of similar MG density (④).

As shown in Figure 2, the unevenness of gland height in the meibography images of *Demodex* group (A) was significantly higher than that in the control group (B). However, there was no significant difference in MG density between them. The total variation, represented by the amplitude of the red line (A④ and B④), can quantitatively measure and distinguish the unevenness of the gland height.

### 3.3 Multivariate Regression Analysis

The total variation is calculated based on the height of each MG; therefore, it may be affected by various MG parameters such as height, density, and quantity, among others. Multivariate regression analysis was used to analyze the factors that impacted the total variation, and the results are shown in Table 3.

**TABLE 3:** Multivariate linear regression analysis of total variation.

Value	Coefficient	*p* value
Eye	−56.65	0.390
Age	4.85	0.066
N (gland number)	38.27	0.0003
H (the total MG height)	−0.16	0.004
D (MG density)	2789.36	0.0002
Lid margin score	−13.89	0.703
Meibum expressibility score	−4.61	0.103
Tear film break-up time	2.16	0.847
Upper lid meiboscore	−42.67	0.504
Lower lid meiboscore	59.07	0.201
OSDI	−0.39	0.834

*p*-value of multivariate regression analysis; OSDI, ocular surface disease index.

Multivariate linear regression analysis revealed that these three factors, N (gland number), H (total MG height), and D (MG density), had an impact on total variation. But the coefficient of H (total MG height) is too small to draw a correlation between H and total variation. Therefore, we corrected the total variation using these two factors (gland number and MG density) and analyzed the statistical results of the corrected total variations as Uneven indexes between the two groups, as shown in Table 4.

**TABLE 4:** Upper lid total variation in *Demodex*-positive and *Demodex*-negative patients.

Parameters		*Demodex* group (*n* = 64)	Control group (*n* = 53)	*p* value	*p* value*
Total variation		927.3 ± 343.1	650.0 (569.5, 865.5)	0.003	0.004
(Range)		(315.0–1698.0)	(152.0–1725.0)		
Uneven index	1. Total variation/N	58.2 ± 19.2	47.9 (34.9, 56.6)	0.001	0.003
	(Range)	(24.5–109.1)	(18.5–115.0)		
	2. Total variation/D	4244.5 (2892.7, 5420.5)	2171.4 (1877.8, 3251.3)	<0.001	<0.001
	(Range)	(1234.3–9988.2)	(975.0–5709.1)		

*p*-value of Mann–Whitney U test or Independent-*t* test for continuous parameters and of χ2 test for categorical parameters; Total variation/N, total variation/gland number; Total variation/D, total variation/MG density; *p values adjusted for age by analysis of covariance

When considering the influence of the gland number and MG density on the uneven degree of MG, we found that there were also statistical differences between the two groups on the two types of Uneven indexes. The average values of total variation (*p* = 0.004), Uneven index 1 (*p* = 0.003), and Uneven index 2 (*p* < 0.001), quantitative measures for the unevenness of MG atrophy, were all significantly higher in the *Demodex* group than in the control group.

### 3.4 Receiver Operating Characteristic Analysis

**FIGURE 3:**
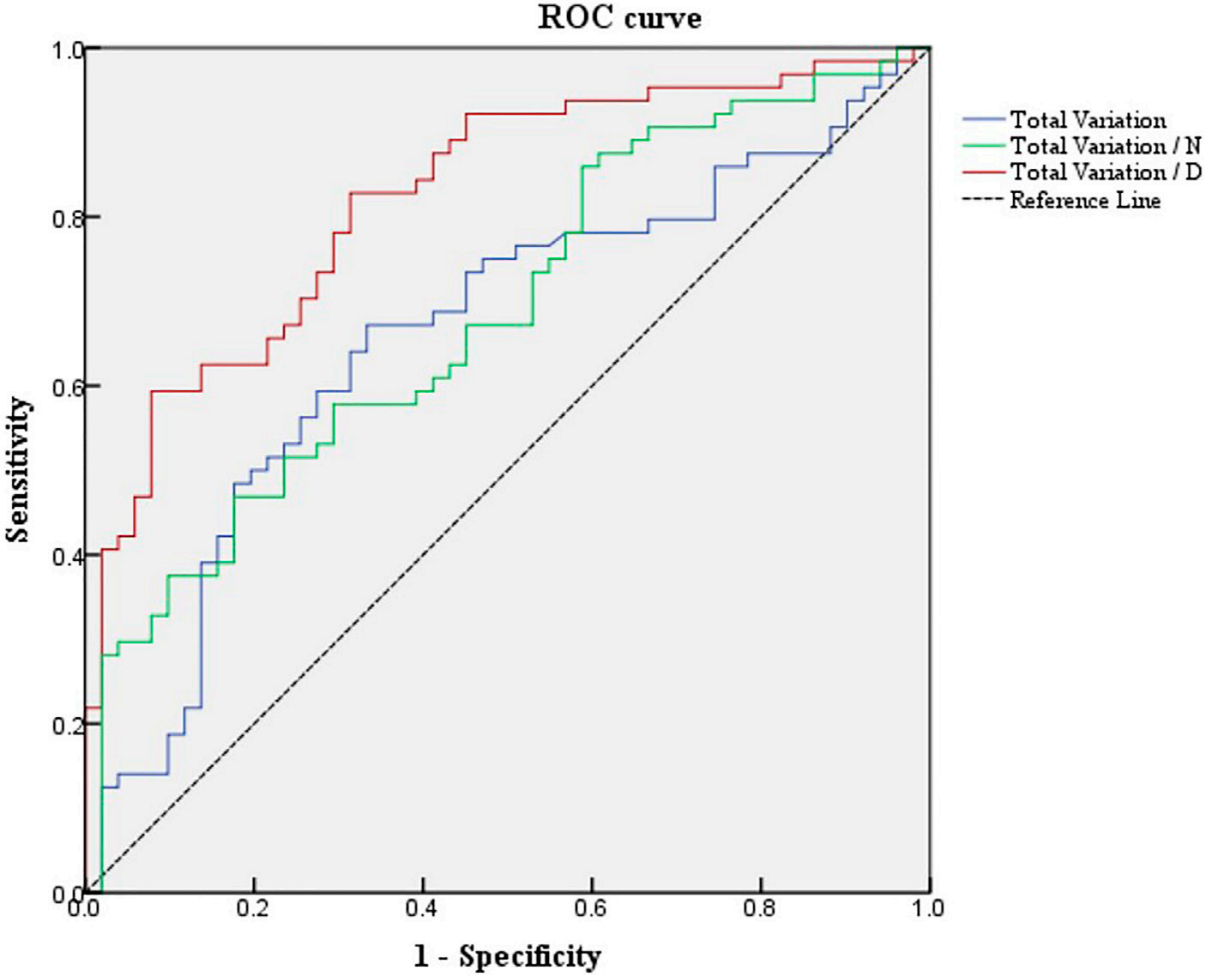
ROC curves of total variation and Uneven indexes differentiate *Demodex* blepharitis subjects from the normal controls. ROC, receiver operating characteristic; Total variation/N, total variation/the number of MGs; Total variation/D, total variation/MG density.

The potential value of total variation and Uneven index was used to discriminate *Demodex* blepharitis individuals from the control group. Based on the multivariate regression analysis results, ROC curves were generated to show the prediction of *Demodex* blepharitis using the variates (Figure 3). There was one type of Uneven index (Uneven index 2) that showed the ability to discriminate *Demodex* blepharitis subjects from the control group.

The area under the curve (AUC) was 0.667 for total variation. The sensitivity and specificity were 67.2% and 66.7%, respectively, at a cut-off value of 778.5.

The AUC was 0.694 for Uneven Index 1 (total variation/N). The sensitivity and specificity were 48.4% and 82.4%, respectively, at a cut-off value of 59.625.

The AUC was 0.822 for Uneven Index 2 (total variation/D). The sensitivity and specificity were 59.4% and 92.2%, respectively, at a cut-off value of 3971.667.

## 4 Discussion

*Demodex* mites are the ectoparasites found most frequently on human skin (Basta-Juzbasić et al., 2002; Yasak et al., 2022). Previous studies found that *Demodex* plays an important role in the pathogenesis of some dermatoses skin, such as papulopustular and/or acneiform lesions without comedones, seborrheic dermatitis-like eruption, perioral dermatitis-like lesions, telangiectasia, bacterial folliculitis, rosacea, and otitis externa (Li et al., 2010; Rather and Hassan, 2014; Aktaş-Karabay E & Aksu-Çerman., 2020). These studies have shown a significantly increased density of D. folliculorum mites in the facial skin of patients with rosacea when compared with control subjects. And *Demodex* may trigger an immune-inflammatory response that stimulates the progression of the infection to the papulopustular stage.

However, *Demodex* mites are not only parasitized widely in the hair follicles of the skin, but also in the hair follicles and meibomian glands of the eyelids; moreover, studies have shown that *Demodex* infestation is one of the factors responsible for chronic blepharitis, conjunctivitis, and MGD (Morfin Maciel, 2003; Kheirkhah et al., 2007; Liang et al., 2010; Hao et al., 2022). Compared with *Demodex*-negative MGD patients, the ocular discomfort and OSDI score were more severe in *Demodex*-infested MGD patients (Lee et al., 2010; Rabensteiner et al., 2019). However, few studies have discussed the correlation between MG morphology and *Demodex* blepharitis.

In this study, we used an AI system based on deep learning to analyze and compare the morphological changes of MGs in *Demodex* blepharitis and normal controls. The AI model in this study was the latest iteration of the CNN model in our previously reported study (Zhang et al., 2022). In our previous study, we found that the changes in height and density of MG in the upper eyelid have more significant correlations with MGD than those in the lower eyelid. This may be because the tarsus of the upper eyelids is more stable and easier to expose; moreover, because the MGs in the upper eyelids are longer, atrophy is relatively obvious. Therefore, this study focused on analyzing MG morphology in the upper eyelids. We found that *Demodex* blepharitis was also associated with uneven characteristic morphological changes in MGs and a significantly shorter height and smaller density. These findings of MG height and density changes are consistent with those of other studies (Liang et al., 2013; Liang et al., 2018; Cheng et al., 2019). However, the overall features of uneven MG morphological changes have not been well described by conventional indexes such as MG height, width, tortuosity, and density in previous studies. Some parameters can be used to indicate how discrete the data are, such as the variance, standard deviation, or coefficient of variation (CV). CV is the ratio of the standard deviation of the original data to its mean of the original data. It can compare the degree of variation between two or more data points with different averages. However, it cannot represent the spatial distribution order of discrete data.

Therefore, we introduced total variation, which includes the spatial relationship between the glands in meibography. The Uneven index based on total variation, a novel index to evaluate the overall features of MG atrophy, was used as a quantitative measure of the unevenness of MG atrophy. In addition, it may serve as a useful digital biomarker for diagnosing *Demodex* blepharitis. As shown in Figure 2, two images with almost the same average height, width, and density were selected from the meibography of the subjects. We found that the amplitudes of the lines connected by the ends of the MGs from the two meibography images were markedly different. The line oscillations in the *Demodex* blepharitis group were significantly higher than those in the control group. When the amplitude was expressed in terms of the total variation, our study found that the total variation and its corrected types, named Uneven index, were significantly higher in the *Demodex* blepharitis group than in the control group (*p* < 0.05). This suggests that there was a significant unevenness in the atrophy of MGs in *Demodex* blepharitis patients. We are the first to use the total variation and Uneven index to evaluate the morphological unevenness of MGs and report the association between *Demodex* blepharitis and the unevenness atrophy of MGs. However, we found that the total variation may be influenced by gland number and degree of gland atrophy, using multivariate regression analysis. We attempted to use these two factors to correct the total variation. In particular, the total variation/D (AUC = 0.822) showed good diagnostic ability. Given the best stability of MG density in this AI system and the best diagnostic efficiency of total variation/D among the two corrected indexes, we recommend total variation/D as the Uneven index for diagnosing *Demodex* blepharitis.

The damage caused by *Demodex* mites involves mechanical damage and results in secondary bacterial infections. *Demodex* mites are considered to be carriers of bacteria, such as *Bacillus oleronius* (Lacey et al., 2007; Szkaradkiewicz et al., 2011; Zhu et al., 2018). A recent study found that the greater the number of *Demodex* mites, the more serious the damage (Cheng et al., 2019). Therefore, the causes of uneven MG atrophy might include different numbers of *Demodex* mites and their activity in different gland ducts, which in turn cause different levels of damage to the glands. Different degrees of gland injury result in different degrees of gland atrophy, and these may be factors that lead to uneven MG atrophy.

The Uneven index can also be calculated by manual labeling without relying on AI. However, it requires much calculation and is difficult to apply in clinical practice. Only with the computer aid can this Uneven index work best. The Uneven index automatically calculated by AI might benefit the prompt of whether patients are accompanied by *Demodex* blepharitis and how much is the *Demodex* effect on the MGs in meibography captured with K5M in our dry eye unit.

This study had some limitations. We did not include non-*Demodex* blepharitis patients as a separate control group. A further control study should recruit the non-*Demodex* blepharitis patients as a control group to further strengthen the evidence that the uneven atrophy of MGs is characteristic of *Demodex* infection. In addition, our current research could not accurately calculate the number of *Demodex* mites in each gland. Therefore, it was impossible to establish a relationship between the degree of atrophy and the quantity of *Demodex* in the individual glands. Further studies are required to investigate the correlation between the number of *Demodex* mites and clinical features as well as morphological changes in MGs.

## 5 Conclusion

In summary, we conclude that uneven morphological changes in MGs are strongly associated with *Demodex* blepharitis. Therefore, the novel characteristic index, the Uneven index, may serve as an important digital biomarker for prompting *Demodex* blepharitis, especially with the aid of AI.

## Data Availability

The original contributions presented in the study are included in the article further inquiries can be directed to the corresponding authors.
